# Medical Society of the County of Erie

**Published:** 1910-12

**Authors:** Franklin C. Gram

**Affiliations:** Secretary


### SOCIETY PROCEEDINGS

### Medical Society of the County of Erie

Reported by FRANKLIN C. GRAM, M. D., Secretary.

THE regular meeting of the Medical Society of the County of Erie was held in the rooms of the Buffalo Society of Natural Sciences, on Monday, October 24, 1910, at 8.30 p. m.

The meeting was called to order by the president Dr. Grover W. Wende.

On motion, the reading of the minutes of the previous meeting was dispensed with, inasmuch as they had been printed in the Buffalo Medical Journal.

The secretary then read the minutes of the Council meetings of October 10 and October 24 respectively, and on motion, they were approved.

Dr. Bennett, Acting Chairman of the Committee on Membership, presented for election to membership in the society, a list of names of applicants as follows who were separately balloted upon and each declared duly elected:

Edith R. Hatch; Andrew C. Callahan, 516 South Park Avenue; William B. May, 149 Glenwood Avenue; J. W. Kiefer, 354 Fillmore Avenue; Harry R. Lohnes, 200 E. Utica Street; James L. Gallagher, 696 Broadway; Warren Z. Dell, 99 Harvard Place; John Tinkler, 1826 Genesee Street; Lester Levyn, 203 Eagle Street; Joseph C. O'Gorman, 1324 Jefferson Street; Alfred Regan, 428 Eagle Street; Anthony J. Cetola, 65 Front Avenue; Joseph Schweitzer, 691 Jefferson Street; Robert S. Taylor, 1518 Fillmore Avenue; P. J. Hurley, 2272 Seneca Street; Hugh J. McGee, 820 Elk Street; Ray A. Edson, 52 Lexington Avenue; John G. Hoeckh, 426 High Street; George Fenn Roberts, 301 Emslie Street; William S. Lynch, 355 N. Division Street; George W. Seitz, 225 E. Genesee Street; Bernard H. Brady, 1865 Bailey Avenue; Stanislaus N. Borowiak, 1303 Broadway; J. C. Clemesha, 329 Franklin Street; Joseph P. Giambrone, 247 Front Avenue; Frank E. Brundage, 131 Allen Street; O. R. Eichel, 205 E. North Street; Herman B. S. Singer, 413 Jefferson Street; John L. Eckel, 400 Forest Avenue; B. Ross Nairn, 362 W. Delavan Avenue; Joseph B. Betts, State Hospital; R. A. Paxton, 83 Putnam Street; George G. Armstrong, 550 Forest Avenue; E. G. Danser, 592 Walden Avenue; John P. Harrison, 400 Forest Avenue; Harry B. Pinkerton, 601 Elmwood Avenue all of Buffalo; B. E. Smith, Angola, N. Y.; Parker G. Bordon, Chaffee, N. Y.; Clarence H. Mackey, 38 E. Main Street, Lancaster; Thomas B. Fowler, 38 Main Street, Springville, N. Y.

The president explained that these forty newly elected members represented a great deal of hard work on the part of the membership committee and those assisting, and that the total number of new members admitted this year was one hundred and forty. The announcement of such an immense addition to the society, with still one more meeting in the current year, was received with approbation.

Dr. Grant, Chairman of the Board of Censors, made report as follows:

*Memorandum Report of the Work of the Board of Censors from June 20, 1910, to October 24, 1910.*

July 27.—Visited Mrs. Candis J. Hall, "metaphysician" at No. 528 Delaware Avenue, Buffalo, and informed her that she was violating the medical law, having a sign "metaphysician" in the window; read the law to her. She claimed to be exempt under the religious tenet clause, said she had a metaphysical diploma and is a member of Rev. Dr. Holmes's Presbyterian Church. Asked time to consult Mr. Locke, her lawyer. Gave her to July 30, and on that day, her son wrote me that the sign would be removed.

July 7.—A trial committee of the State Board of Examiners met in Buffalo to hear and determine upon the charges preferred to the Regents by the Chairman of the Board against Dr. Hugo Schmidt and Dr. William R. Little of Buffalo, for aiding and abetting violation of the cocaine law and otherwise violating the medical practice statute. The trial committee reported to the Regents that Schmidt was guilty and on September 22, 1910, his license to practise was revoked. Dr. Little's case was put over for final determination until the meeting of the Regents on October 27, 1910.

September 14.—The "Neal Institute" case, mentioned in the last report should have called for trial on this date but City Court Clerk could not find "information and warrant," a rather curious condition of affairs. The defendant's attorney, Mr. Abbott, also made claim that as the original corporation had become defunct by filing new papers as a Therapeutic Company, there is no jurisdiction as there is no corporation to proceed against under old information and warrant. This was apparently a subterfuge but the Assistant District Attorney advised that we drop the case and if possible proceed anew under new evidence, which evidently will be hard to get. The case remains in abeyance.

September 10.—Theodore Vogelgesang of No. 442 Jefferson Street, was arrested on a warrant sworn out by the chairman and on September 14, 1910, he was found guilty of practising medicine illegally and fined $50.00. Claim was made on the City Clerk for the amount of this fine and it has been paid.

October 8.—An alleged corporation known as "Dr. Linn's Museum," "Gallery of Scientific Wonders," "Buffalo Medical

Clinic." "Dr. Stevens, Dr. Linn's Successor,". No. 203 Main Street, Buffalo, was arrested in the person of Stevens, claiming to be manager. Also one, John Doe, arrested in the person of Dr. Hancock, who is employed there as a physician and practising under a name other than his own, at least no one knew his name until after arrest. These parties were arraigned in Court October 10, and cases put over until October 25, 1910.

Several other cases have been under investigation; one, a fellow purporting to be the son of Dr. William Douglas, of Fort Erie, Ontario (who denies having a son), was on the eve of arrest for filling prescriptions signed "Dr. Douglas" at Hilligas's Drug Store, Niagara and Carolina Streets, Buffalo, when he must have received a tip and absconded. I hear now that he has located at Seattle, Washington.

A bonesetter and all round medical fakir among the Polish people of Buffalo, one Martin Rybcysnski of No. 408 Sweet Avenue was arrested October 14, and October 17 convicted, fined $50.00, which he failed to pay and was sent to the Erie County Jail for fifty days.

A warrant for $50.00 fine against Anna Wrzesinska convicted for illegal practice January 21, 1910, was paid July 25, and turned over to Dr. Lytle, Treasurer of the society. One more fine of $50.00 is due and will be paid very soon.

In the trial and prosecution of the above cases, your chairman has been ably assisted by Attorney Charles E. Doane, who has devoted a considerable portion of his time to this work.

Respectfully submitted,

For the Board of Censors,

John H. Grant,

*Chairman.*

The foregoing report was accepted and ordered filed, and a vote of thanks given to the chairman and the board for the excellence of the work.

The president announced that the proposed amendment to the by-laws, which had been offered at a previous meeting and notice of which had been sent to the members, was now before the society.

Dr. Wall moved that action on the amendment to the by-laws, offered at the June meeting, be postponed to the next meeting.

After some discussion, Dr. Wall withdrew his motion and moved, as a substitute, the following:

"Make present Sec. 1, Sec. 3. And have Sec. 1 read—"Sec. 1. This society shall be governed by its by-laws, rules and resolutions, by the constitution, by-laws, rules and resolutions of the Medical Society of the State of New York, and by the Principles of Ethics of the American Medical Association."

The substitute motion of Dr. Wall as above was adopted.

Dr. Wall then offered the following amendments to the by-laws:

*Proposed Amendments to the By-Laws.*

Amend Chap. 1, Sec. 1 to read—"This society is an integral part of the Medical Society of the State of New York, being composed of those members who reside in Erie County, and its name and title shall be The Medical Society of the County of Erie."

Amend Chap. II, Sec. 5—"Insert after the word 'signing' a statement that he will be governed by."

Amend Chap. V, Sec. 2—Insert between the word "Society" and "Appropriated" "as authorized by these by-laws and as"

Amend Chap. V, Sec. 3 to read "It shall submit at each regular meeting a report of the work done."

Amend Chap. VII, Sec. 4. Add after "Co-operate with" "and take no action on State legislation or appointments until approved."

Amend Chap. VIII, Sec. 2. Substitute for the present section—"In case a delegate cannot attend any meeting the council shall appoint an alternate and if any representative fail to appear the other delegates shall choose a member present to act for him."

Amend Chap. XI, Sec. 1. Substitute for words "as arranged" "any regular meeting may be held earlier or later when considered desirable."

Amend Chap. XI, Sec. 5 to read "The order of business when not arranged by the council and printed in the notice of the meeting shall be."

Amend Chap. XV, Sec. 1. Insert between words "been" and "sent" "approved by the council of the state society and advice of a proposed change."

Amend Chap. XV, Sec. 2 to read "Any by-law except Chap. XV, Sec. 1, may be suspended for any meeting."

Under the rules these amendments must lie on the table until next meeting, when they may be voted upon.

This closed the business part of the session and the president introduced Dr. William T. Shanahan, Superintendent of Craig Colony for Epileptics, Sonyea, N. Y., as the speaker of the evening.

Dr. Shanahan presented a very carefully prepared paper together with some charts representing the work at Sonyea, and spoke of the methods in use at the Craig Colony. The paper elicited very spirited discussion participated in by Dr. A. W. Hurd, Superintendent of Buffalo State Hospital, Drs. Crego, Putnam, Benedict, VanPeyma and others.

Dr. Shanahan then closed the discussion after which he was given a hearty vote of thanks for his very interesting paper.

A collation followed and a social hour was spent by most of the more than 150 physicians who attended the meeting.

